# Case report: A case of hypocalcemic cardiomyopathy with non-reversible cardiac fibrosis

**DOI:** 10.3389/fcvm.2023.1166600

**Published:** 2023-08-21

**Authors:** Ying Xie, Jing Yang, Kun Li, Fang Liu, Yi Yi, Ping Zhang

**Affiliations:** ^1^ Department of Cardiology, Beijing Tsinghua Changgung Hospital, School of Clinical Medicine, Tsinghua University, Beijing, China; ^2^ Department of Radiology, Beijing Tsinghua Changgung Hospital, School of Clinical Medicine, Tsinghua University, Beijing, China

**Keywords:** hypocalcemic cardiomyopathy, heart failure, cardiac fibrosis, non-reversible, thyroidectomy

## Abstract

Hypocalcemic cardiomyopathy is a rare etiology of heart failure. It is considered highly reversible with a relatively favorable prognosis. This case reports a 52-year-old housewife diagnosed with hypocalcemic cardiomyopathy who presented with acute decompensated heart failure and hypocalcemia symptoms with a history of thyroidectomy. Shortness of breath and edema were relieved after diuresis and prompt electrolyte correction. The left ventricular ejection fraction increased from 27% to 53%, and the left ventricular end-diastolic dimension shortened from 58 to 50 mm in echocardiographic re-examinations, while repeat cardiac magnetic resonance imaging revealed evidence of non-reversible cardiac fibrosis after 1-year follow-up. Cardioprotective agents with close follow-ups were called for in this entity of patients.

## 1. Introduction

Heart failure with reduced ejection fraction is one of the major health problems worldwide. Within different etiologies, hypocalcemia is one of the causes considered to be relatively benign and reversible, with prompt correction of electrolyte disturbance, diuretic treatment, and other guideline-directed medical therapies for heart failure. However, the recovery of underline pathophysiological change is not clear. Here, we present a case with heart failure and prominent hypocalcemia, who had rapid improvement after replacement therapy. However, cardiac magnetic resonance (CMR) imaging showed persistent evidence of cardiac fibrosis, which may indicate hypocalcemia-induced non-reversible myocardium damage.

## 2. Case description

A 52-year-old housewife was admitted to our hospital with the chief complaint of exercise intolerance and severe shortness of breath for 1 month. She received a subtotal thyroidectomy for a goiter 11 years ago. Since then, therapy with levothyroxine and calcium supplement for hypoparathyroidism has been prescribed. The thyroid function or serum calcium level was not tested without regular follow-ups. She had suffered occasional hand and arm twitching after thyroidectomy for 10 years. The patient did not have a medical history of hypertension, coronary heart disease, and diabetes mellitus nor a family history of cardiomyopathy or sudden cardiac death.

Upon admission, a physical examination showed tachypnea with a respiratory rate of 28 breaths per minute and tachycardia with a heart rate of 100 beats per minute. The obvious distended jugular vein and lower extremity edema were noted. Respiratory crepitations were heard in bilateral base lungs, and a neurology examination revealed positive Chvostek's sign indicating hypocalcemia.

**Figure 1:**
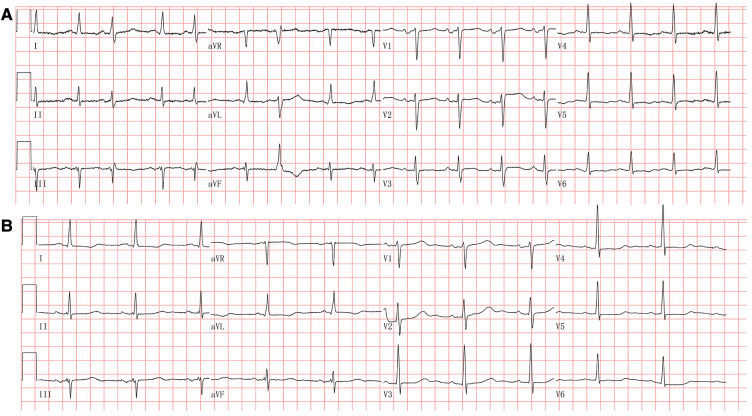
(**A**) Electrocardiogram at admission. The electrocardiogram showed sinus tachycardia and multiple premature atrial complexes with aberrant conduction. The QT interval was prolonged with a corrected QT interval at 523 ms. (**B**) Electrocardiogram at the 3-month follow-up. The electrocardiogram showed sinus rhythm with a corrected QT interval at 491 ms.

The electrocardiogram showed a prolonged heart rate corrected QT interval (QTc) of 530 ms primarily by prolonging the ST segment and multiple premature atrial complexes with aberrant conduction (Figure 1A). The chest X-ray detected pulmonary edema and cardiac enlargement. Laboratory analysis at admission revealed elevated levels of N-terminal prohormone of brain natriuretic peptide (NT-proBNP), significant hypocalcemia (ionized calcium 0.45 mmol/L, normal range 1.12–1.32), hypomagnesemia, and hypokalemia. The parathyroid hormone (PTH) was 13.63 ng/L (normal range 15.0–65.0), and thyroid-stimulating hormone (TSH) increased to 15.608 mIU/L (normal range 0.55–4.78) (Table 1). Transthoracic echocardiography showed cardiac dilatation, and the left ventricular end-diastolic diameter (LVEDD) was enlarged to 58 mm. Impaired left ventricular function with left ventricular ejection fraction (LVEF) was only 29%. Coronary angiography revealed a non-obstructive lesion in the left anterior artery. She declined the gene test due to the high cost.

**Table 1:** Biochemistry and hormonal values.

	On admission	At discharge
Glycemia (4.11–5.89 mmol/L)	7.93	4.76
ALT (7–40 U/L)	35.5	16.7
AST (13–35 U/L)	41.8	18.8
Total bilirubin (0–21 μmol/L)	31.03	28.1
Direct bilirubin (≤8 μmol/L)	15.91	12.7
Albumin (35–52 g/L)	36.4	34.8
Creatinine (45–84 μmol/L)	148.8	120.3
Sodium (135–145 mmol/L)	140.83	135.97
Potassium (3.5–5.5 mmol/L)	3.11	4.35
Total serum calcium (2.15–2.50 mmol/L)	1.09	2.06
Ionized calcium (1.12–1.32 mmol/L)	0.45	1.08
Serum magnesium (0.66–1.07 mmol/L)	0.52	0.80
NT-proBNP (0–125 pg/ml)	12,258	2,483
CK-MB (0–4.88 ng/ml)	10.46	3.5
Troponin T (0–0.014 ng/ml)	0.029	0.016
Serum cholesterol (<5.2 mmol/L)	2.66	—
HDL-C (>1.0 mmol/L)	0.57	—
LDL-C (<3.4 mmol/L)	1.97	—
Triglycerides (0–1.7 mmol/L)	0.90	—
Parathormone (15–65 pg/ml)	13.63	—
TSH (0.55–4.78 mIU/L)	15.608	—
25-OH-vitamin D (30–100 ng/ml)	17.28	—

ALT, alanine aminotransferase; AST, aspartate aminotransferase; CK-MB, creatine kinase-MB; HDL-C, high-density lipoprotein-cholesterol; LDL-C, low-density lipoprotein-cholesterol.

**Figure 2:**
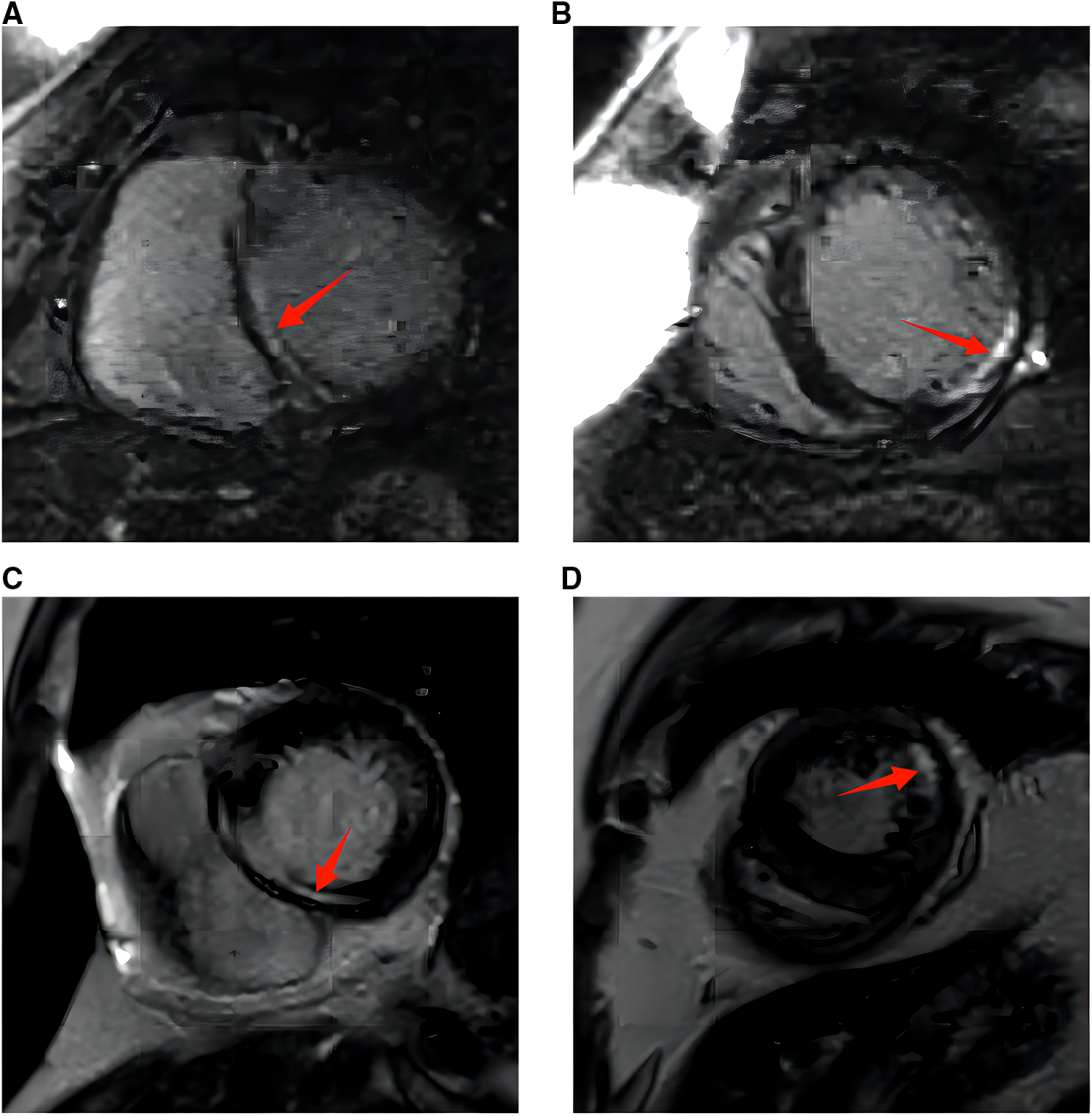
(**A**) Cardiac magnetic resonance imaging with gadolinium at diagnosis. The red arrow marks late gadolinium enhancement in the interventricular septum (bright liner enhancement). (**B**) Cardiac magnetic resonance imaging with gadolinium at diagnosis. The red arrow marks late gadolinium enhancement in the inferior and lateral walls of the left ventricle [or left ventricular something (bright liner enhancement)]. (**C**) Cardiac magnetic resonance imaging with gadolinium at 10 months. The red arrow marks late gadolinium enhancement in the interventricular septum (bright liner enhancement). (**D**) Cardiac magnetic resonance imaging with gadolinium at 10 months. The red arrow marks late gadolinium enhancement in the inferior and lateral walls of the left ventricle (bright liner enhancement).

A primary diagnosis of hypocalcemic cardiomyopathy (HC) was made in view of the congestive heart failure, the potentially long-existing untreated hypocalcemia resulting from hypoparathyroidism, and the lack of evidence of other etiologies including hypertension, valvular, congenital, or ischemic heart disease. The subject was treated with oxygen inhalation, intravenous diuresis, and electrolyte supplements. Calcitriol was simultaneously started along with guideline-directed medical therapy (GDMT), including metoprolol and perindopril, which were gradually titrated to the maximal tolerated dosage. She was intolerant to spironolactone treatment because of acute kidney injury, with serum creatinine increasing from 96 to 221 µmol/L in 1 week. Serum calcium, magnesium, and potassium were normalized within 2 weeks of initiation. Clinical symptoms were relieved significantly following optimal medical therapy. After stabilization, the patient underwent CMR imaging, which showed signs of cardiac fibrosis in the septal, lateral, and inferior walls of the left ventricle after late gadolinium enhancement (LGE) (Figures 2A,B). The 24-h ambulatory electrocardiographic recording showed isolated atrial premature beats, paroxysmal atrial tachycardia, and isolated ventricular premature beats.

Repeated electrocardiography at the 3-month follow-up showed QTc shortened to 491 ms (Figure 1B). Transthoracic echocardiography at posthospitalization follow-up visits found LVEF improved to 34% at 3 weeks and 53% at 3 months. LVEDD decreased to 50 mm at 3 months and remained to be enlarged after that (Table 2). CMR imaging was conducted at the 1-year follow-up to review fibrosis size, and it exhibited similar positive LGE in the same area, as shown in Figures 2C,D. The patient remained symptom-free after discharge. She underwent regular ambulatory electrocardiographic monitoring tests every 3 months during follow-ups, which revealed similar findings to previous results and showed isolated premature ventricular complexes originating from the right ventricular outflow tract, without ventricular tachyarrhythmia.

**Table 2:** Diagnostic and follow-up transthoracic echocardiography measurements.

Date	LA (mm)	LVEDD (mm)	IVSd (mm)	LVEF (%)
24 May 2019	42 × 68 × 49	58	10	29
15 June 2019	45 × 68 × 54	59	10	34
22 July 2019	38 × 66 × 51	55	10	41
6 September 2019	43 × 65 × 53	50	9	53
25 March 2020	43 × 63 × 48	52	10	49
22 December 2021	46 × 62 × 49	53	10	47
29 July 2022	41 × 59 × 45	52	10	47

LA, left atrial; LVEDD, left ventricular end-diastolic diameter; IVSd, Interventricular septal diameter; LVEF, left ventricular ejection fraction.

## 3. Discussion

### 3.1. Clinical presentation, treatment, and outcomes of HC

Hypocalcemia is a rare cause of heart failure with reduced ejection fraction (HFrEF). Postsurgical hypoparathyroidism is the most common cause of hypocalcemia. A recent literature review summarized 61 case reports and described the most common causes of HC as being primary hypoparathyroidism (50%) and post-thyroidectomy hypoparathyroidism (26%) (1). The clinical manifestations of hypocalcemia encompass a diverse range of symptoms and signs that impact virtually all major physiological systems, including the cardiovascular, neurological, renal, and respiratory systems. The presentation ranges from asymptomatic to severe, life-threatening seizures, heart failure, or laryngospasm, depending on the severity and duration of hypocalcemia (2). Patients with few symptoms may neglect and leave hypocalcemia untreated for a long time, such as in our case. The duration of hypocalcemia to the onset of heart failure varies in different patients. A review summarized cases with hypocalcemia secondary to thyroidectomy and found that the median duration for the development of HC was 10 years, with a wide range of 1.5 months to 36 years (1). This difference may be related to the severity of hypocalcemia and the susceptibility to cardiomyopathy in individual patients, including genetic variations.

The primary treatment of hypoparathyroidism and resulting hypocalcemia includes activated vitamin D analogs plus calcium supplements to raise the calcium level to the lower normal range while avoiding hypercalciuria (3, 4). Cardiomyopathy and heart failure should be managed as standard treatment per societal guidelines (5, 6). Most HC cases reported significant LVEF improvement after optimal heart failure treatment and serum calcium correction. Many literature works have mentioned that the symptoms and echo findings of HC may improve rapidly with the guideline-directed medical therapy of heart failure and correction of hypocalcemia (7–9). The improvement response of heart failure symptoms might occur as short as a few days, and a significant improvement from NYHA class IV to class I could happen within 1 week (9, 10). Very few cases (5%) showed resistance to therapy and had non-reversible cardiac dysfunction (1). Authors have attributed the reasons for poor response to the therapies to poor compliance, underlying cardiomyocyte degeneration, or the development of cardiac fibrosis (11).

### 3.2. Underlying cardiac fibrosis in HC

The literature has documented the presence of cardiac fibrosis in patients with HC. Altunbas et al. have presented two cases of HC resulting from hypoparathyroidism that were left untreated for extended periods, i.e., 19 and 12 years, respectively. The patients underwent endomyocardial biopsy, which revealed hypertrophic and atrophic changes in conjunction with increased fibrous tissue. The patients showed clinical improvement after treatment with calcium supplements and calcitriol, although no follow-up data were available (12). Recently, CMR has gained widespread acceptance as a diagnostic tool for detecting fibrosis. CMR imaging was performed on our patient, and the results revealed the presence of cardiac fibrosis, which is consistent with the two cases reported by Altunbas et al. (12).

The development of myocardial fibrosis may be related to the duration and severity of hypocalcemia. Our patient underwent surgery for hypoparathyroidism 11 years ago, implying prolonged untreated hypocalcemia. Some researchers have also employed CMR imaging in patients with hypoparathyroidism; however, they have not been able to detect LGE. For instance, Valek et al. presented a case of a 26-year-old woman with HC, where CMR imaging did not reveal any evidence of fibrosis (1). The patient had only been afflicted with hypoparathyroidism for a year. Two other case studies also reported negative findings of cardiac fibrosis in CMR imaging, and their duration of hypocalcemia was 6 and 5 years, respectively (8, 9).

The detailed mechanism underlying the formation of cardiac fibrosis in HC remains unknown. In general, cardiomyocyte death and/or mechanical, ischemic, or metabolic injury may activate fibroblasts and stimulate the formation of fibrosis (13). Calcium plays an important role in initiating electric activation, ion channel gating, epinephrine-induced glycogenolysis, and excitation and contraction coupling. The myocardial electrophysiological feature and contractility change during acute hypocalcemia have been demonstrated in other study models (14). Prolonged hypocalcemia might have resulted in myocardial injuries and subsequent fibrosis. Further investigations are needed to explore the relationship between HC and myocardial fibrosis.

Cardiac fibrosis is thought to be related to unfavorable outcomes resulting from cardiac dysfunction, reduced perfusion, and propensity to arrhythmias (13). Although the patient in our case experienced rapid symptom relief and improvement in left ventricular function, the presence of underlying fibrosis suggests that this improvement might be a case of “myocardial remission” rather than “myocardial recovery.” The risk of heart failure recurrence and arrhythmias in the long term persists. This highlights the importance of ongoing treatment with cardioprotective agents and close monitoring through regular follow-up evaluations. In the event of syncope or the manifestation of symptoms associated with ventricular arrhythmias during subsequent follow-up sessions, it may be advisable to conduct an assessment employing an implantable loop recorder or electrophysiological study (15).

## Data Availability

The original contributions presented in the study are included in the article/Supplementary Material, further inquiries can be directed to the corresponding author.
